# Developing Synthetic Full-Length SARS-CoV-2 cDNAs and Reporter Viruses for High-Throughput Antiviral Drug Screening

**DOI:** 10.3390/v18010044

**Published:** 2025-12-27

**Authors:** Megha Rohamare, Nidhi Kaushik, Juveriya Qamar Khan, Mahrokh Balouchi, Joaquin Lopez-Orozco, Robert Kozak, Tom C. Hobman, Darryl Falzarano, Anil Kumar, Joyce A. Wilson

**Affiliations:** 1 Department of Biochemistry, Microbiology, and Immunology, University of Saskatchewan, Saskatoon, SK S7N 5E5, Canada; mrr968@usask.ca (M.R.); hiq645@mail.usask.ca (N.K.); juveriya.khan@usask.ca (J.Q.K.); mab088@mail.usask.ca (M.B.); 2 Vaccine and Infectious Disease Organization, University of Saskatchewan, Saskatoon, SK S7N 5E3, Canada; darryl.falzarano@usask.ca; 3 Department of Cell Biology, Faculty of Medicine & Dentistry, University of Alberta, Edmonton, AB T6G 2H7, Canada; lopezoro@ualberta.ca (J.L.-O.); tom.hobman@ualberta.ca (T.C.H.); 4 Biological Sciences, Sunnybrook Research Institute, Toronto, ON M4N 3M5, Canada; rob.kozak@sunnybrook.ca; 5 Shared Hospital Laboratory, Toronto, ON M1E 4B9, Canada; 6 Department of Laboratory Medicine and Pathobiology, University of Toronto, Toronto, ON M5S 1A1, Canada; 7 Department of Medical Microbiology & Immunology, Faculty of Medicine & Dentistry, University of Alberta, Edmonton, AB T6G 2E1, Canada; 8 Li Ka Shing Institute of Virology, University of Alberta, Edmonton, AB T6G 2E1, Canada; 9 Department of Veterinary Microbiology, University of Saskatchewan, Saskatoon, SK S7N 5B4, Canada

**Keywords:** SARS-CoV-2, coronavirus, reporter, luciferase, COVID-19

## Abstract

The continuing spread of SARS-CoV-2 and the associated morbidity and mortality, especially in vulnerable populations, highlight the need for the development of antiviral therapeutics. Reverse genetics systems and reporter viruses are valuable for antiviral screening by simplifying methods to detect and quantify virus infections. This study aimed to generate wild-type and Nluc reporter full-length SARS-CoV-2 molecular clones and viruses as tools for high-throughput antiviral assays. The large SARS-CoV-2 genome (~30 kb) makes cDNA cloning and virus rescue technically challenging, so we opted to use cDNA chemical synthesis services to generate full-length wild-type and reporter Delta and Omicron clones. Clone-derived Delta and Omicron wild-type and reporter viruses were successfully rescued and showed replication kinetics comparable to patient-derived isolates. Nluc reporter viruses displayed stable luciferase expression that correlated with viral titres, supporting their reliability as replication substitutes. Antiviral assays measuring replication inhibition by Remdesivir, Molnupiravir, and Nirmatrelvir, based on Nluc expression, yielded IC_50_ values and selectivity indices consistent with published ranges. Finally, Delta Nluc viruses replicated in primary human bronchial epithelial cells, demonstrating the application of clone-derived viruses in physiologically relevant models. The SARS-CoV-2 cDNA clones and Nluc reporter viruses derived from DNA synthesis services provide a rapid, scalable reverse genetics platform for generating new viruses and developing assays to rapidly assess antiviral compounds against current and emerging SARS-CoV-2 variants or coronaviruses that may emerge in the future.

## 1. Introduction

Severe Acute Respiratory Syndrome Coronavirus-2 (SARS-CoV-2) emerged in late 2019, causing the global pandemic of Coronavirus disease 2019 (COVID-19). SARS-CoV-2 belongs to the genus betacoronavirus of the family Coronaviridae [1]. Patients with COVID-19 exhibit mild to moderate flu-like symptoms, and about 15% of individuals may advance to severe pneumonia, with around 5% experiencing acute respiratory distress syndrome (ARDS), septic shock, or multiple organ failure and death [2,3]. As of November 2025, the WHO COVID-19 dashboard has reported over 778 million cases and over 7 million deaths (https://covid19.who.int/). The rapid development of vaccines, including mRNA vaccines (Pfizer-BioNTech and Moderna) and protein subunit vaccines (Novavax), significantly reduced the burden of disease. Through the pandemic, the virus evolved to escape vaccine-induced immunity and adapt to infection in humans, leading to the emergence of variants like Omicron. Omicron continues to evolve and initiate new waves of infection [4], and while the currently spreading viruses cause severe disease less frequently, there remain few antiviral treatment options for those who do develop severe disease.

One method that facilitates the development of antiviral agents has been the creation of recombinant reporter viruses that express fluorescent or bioluminescent proteins. Reporter protein expression during a virus infection allows for the development of simple assays to measure virus replication that are amenable to high-throughput drug screening methods. Both eGFP and NanoLuc (Nluc) reporters have distinct advantages for monitoring viral replication. eGFP reporter viruses enable direct visualization of infected cells and subcellular localization, making them useful for qualitative imaging and for tracking infection at the single-cell level. However, because eGFP is cell-associated, it requires cell lysis or microscopy for quantification, which can be labour-intensive and limits longitudinal studies in the same culture. In contrast, Nluc is a small, secreted luciferase that produces luminescent signals with minimal background, high sensitivity, and a broad dynamic range [5,6]. Its activity can be measured directly in the culture medium, enabling non-destructive, longitudinal monitoring of viral replication over time. Additionally, Nluc’s small size contributes to greater genetic stability and reduces the risk of reporter-associated attenuation. Together, these features make Nluc reporter viruses suitable for sensitive, quantitative, and high-throughput studies, and complement the use of eGFP reporter viruses for imaging and qualitative analysis. However, the development of reporter viruses requires a viral reverse genetic system; a DNA copy of the viral genome which can be manipulated and then used to generate an infectious virus, and introduction of the reporter gene in a location that does not compromise viral fitness.

Typically, reverse genetic systems for positive-sense single-stranded RNA viruses are relatively simple to generate. They involved converting the RNA genome into a cDNA copy, amplifying it on a bacterial plasmid, and then using the cDNA to synthesize an authentic copy of the viral RNA genome. The viral genomes of positive-sense single-stranded RNA viruses are infectious, so they will initiate a viral infection if produced within or transfected into permissive cells. However, the SARS-CoV-2 genome is approximately 30 kb long, one of the largest viral RNA genomes, making the generation of a full-length cDNA copy cumbersome, and its large size and specific sequences often lead to plasmid instability when grown in bacterial cells. Several methods have been developed to overcome these difficulties inherent to coronavirus reverse genetic systems [7], including the use of cDNA fragments that are annealed in vitro to generate a cDNA and genomic RNA [8,9], recombination systems in yeast [10], and the use of single copy bacmids to facilitate the growth of large viral cDNAs in bacteria.

This study focuses on the design and construction of full-length SARS-CoV-2 variant clones and Nluc reporter gene versions in bacmid plasmids by using commercial-sourced DNA synthesis and assembly services provided by the company Telesis Bio [11,12]. We successfully generated full-length cDNA clones of the Delta and Omicron wild-type variants as well as reporter versions that express Nluc. The cDNA clones were used to generate viral genomic RNAs, and the RNA was used to initiate viral infections in a Biosafety Level 3 (BSL3) laboratory. In addition, we generated viruses from cDNAs that had been amplified in bacteria. We confirmed the authenticity of the clone sequences, clone-derived viruses, and their fitness in infected cells. The reporter gene expression correlated with virus replication, validating the utility of the reporter virus in antiviral assays. Our findings indicate that DNA synthesis is a viable method to rapidly generate coronavirus cDNAs and recombinant viruses, and that Nluc-reporter Omicron and Delta viruses allow for rapid viral replication evaluation and provide an easy means of monitoring virus replication to expedite development and assessment of antiviral therapies targeting SARS-CoV-2.

## 2. Materials and Methods

Cell lines—All the cells were maintained at 37 °C with 5% CO_2_. African Green monkey kidney cells Vero (ATCC CCL-81) were grown in DMEM growth media (Sigma, Markham, ON, Canada, #D5796) supplemented with heat-inactivated 10% Fetal Bovine Serum (FBS; Gibco, ON, Canada, #12483020) and added 1% Pen-strep solution (Gibco, ON, Canada, #15140122). Normal Human Bronchial Epithelial Cells (NHBE) obtained from Lonza (#CC-2540) were grown in BEBM™ Bronchial Epithelial Cell Growth Basal Medium (BEBMTM Medium, Clonetics Airway Epithelial Cell Systems) with Retinoic Acid (Lonza, Basel, Switzerland #:CC-2540). The BEBM media was supplemented with contents of the BEBMTM SingleQuotsTM Kit (#CC-3171 containing Bovine Pituitary Extract [BPE], Hydrocortisone, Human Epidermal Growth Factor [hEGF], Epinephrine, Transferrin, Insulin, Retinoic Acid, Triiodothyronine, and Gentamicin/Amphotericin-B) according to the manufacturer’s instructions.

Construction of SARS-CoV-2 molecular clones—We generated full-length and luciferase reporter clones of SARS-CoV-2 Delta and Omicron variants for this study. The sequences used to generate Delta WT molecular clones were obtained from the GISAID (Global Initiative on Sharing All Influenza Data) database, a global repository for genomic data related to influenza and viruses, including SARS-CoV-2. The SARS-CoV-2 Delta WT clone is based on the EPI SET Identifiers-3162224, 3162387, and 3162814. Omicron WT was constructed based on the GISAID database EPI SET Identifiers 249358, 2B95A89, 32703755, 211129-001, VM21044713-1, RIVM-71082, K032193, K032194, K032221, N21665, N21676. Full-length SARS-CoV-2 cDNA clones were generated by Telesis Bio (formerly Codex DNA Inc., San Diego, CA, USA; #SC2-FLSG-3333) using the BioXp^®^ system, an automated benchtop platform for high-fidelity DNA synthesis, assembly, and cloning. We designed the desired DNA sequences digitally using SnapGene (GSL Biotech, Chicago, IL, USA) and submitted them to Telesis Bio for synthesis. Constructs were automatically synthesized, assembled, error-corrected, amplified, and delivered ready-to-use for downstream experiments. Full-length wild-type (WT) and reporter clones of Delta and Omicron cDNA were inserted into a low-copy bacterial artificial chromosome (BAC) plasmid (pCC1BAC) for further molecular cloning and virus rescue. The BAC was synthesized by Telesis Bio. The bacmids were transformed into Lucigen TransforMax™ EPI300™ Electrocompetent E. coli cells (Lucigen, Biosearch Technologies, Middleton, WI, USA # EC300110). The EPI300™ cells carry a mutant trfA gene under tightly regulated control of an inducible promoter for plasmid copy number control and high transformation efficiency with clones of all sizes [13]. Transcription of full-length RNA-launched WT viruses was driven by the T7 promoter sequence upstream of SARS-CoV-2 virus 5′UTR and a T7 terminator, polyA sequence and Hepatitis Delta Virus (HDV) ribozyme sequence downstream of the 3′UTR [14]. To generate full-length luciferase reporter clones, a gene expressing Nluc was inserted downstream of the nucleocapsid (N) transcription regulatory sequence (TRS) in frame with the N, followed by the porcine teschovirus-1 2A peptide (27 bases) to separate Nluc and N during translation [15]. The plasmid pcDNA-N used for making N mRNA was a gift from Dr. Qiang Liu [11,12].

Plasmid amplification—For amplification of the Delta WT bacmid, we followed the previously established protocol as described [16]. Note that specific bacterial growth conditions are required to optimize the stability of the bacmids. The plasmid DNA extraction and purification were performed using the NucleoBond Xtra Maxi-prep kit for transfection-grade plasmid DNA (MACHEREY-NAGEL GmbH & Co. KG, Düren, Germany #740414.50).

**Figure 1:**
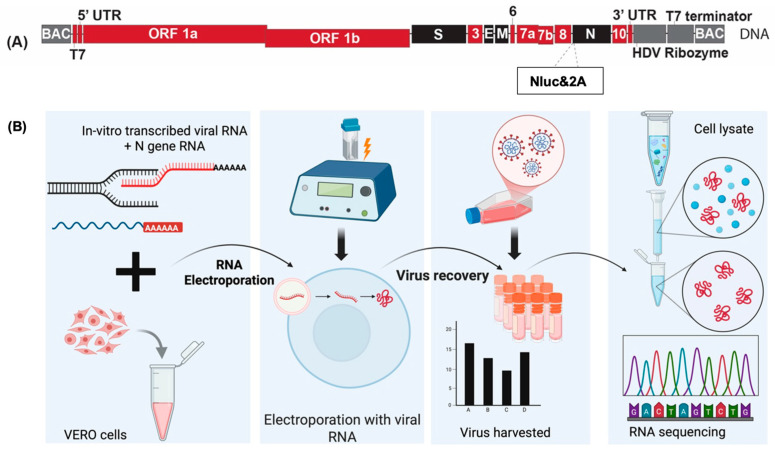
Schematic overview of SARS-CoV-2 clone generation and analysis. (**A**) Generic SARS-CoV-2 genome organization diagram. (**B**) Schematic illustration of viral RNA synthesis and electroporation in Vero cells. Viral RNA was synthesized in vitro from the bacmid DNA template using IVT. The synthesized RNA was then electroporated into Vero cells for virus generation and propagation. The virus was passaged 5 times, and viral titres were monitored at each passage. Viral RNA was sequenced at every passage to assess sequence stability. These were performed in a single biological replicate. (Created in BioRender. Rohamare, M. (2025) https://BioRender.com/fpa0vs3).

In vitro transcription—We created and analyzed full-length and reporter SARS-CoV-2 viruses for both Delta and Omicron variants. The method used to generate viruses from synthetic virus clones is illustrated in Figure 1. In vitro transcription (IVT) reactions to generate full-length viral RNA were performed using the mMESSAGE mMACHINE™ T7 Transcription Kit (Invitrogen, Carlsbad, CA, USA, #AM1344) and 1.5–2 μg of purified DNA templates (Figure 1A). The DNA does not require linearization due to the ribozyme and T7 terminator downstream of the 3′UTR. To purify the RNA, we used phenol-chloroform extraction and precipitation following the protocol described previously [11,17]. N gene RNA was in vitro transcribed from 1 µg of pcDNA-N that had been linearized with Xba I (New England Biolabs, Ipswich, MA, USA #R0145S) for 1 h at 37 °C, followed by Mung bean Nuclease (New England Biolabs, #M0250S) treatment for 1 h at 37 °C. The linear DNA was then purified and precipitated to be transcribed using the mMessage mMachine^TM^ T7 Ultra kit (Invitrogen #AM1345) according to the manufacturer’s instructions. Purified viral genomic and N-gene RNA was measured using a Qubit^TM^ RNA High Sensitivity kit (Invitrogen, Carlsbad, CA, USA #Q32852) as per the manufacturer’s protocol.

Electroporation and virus generation from IVT RNA—Purified SARS-CoV-2 full-length viral genomic RNA and N-gene mRNA derived from the IVT reactions were co-electroporated into Vero cells using previously established methods (Figure 1B) [11,17]. In brief, the cells were washed with Dulbecco’s phosphate-buffered saline (DPBS) (Gibco™, Grand Island, NY, USA #14190144), trypsinized with Trypsin-EDTA (0.25%) (Gibco™, Grand Island, NY, USA #25200056), and washed with DPBS before resuspending at 1.5 × 10^7^ cells/mL in Ingenio^®^ Electroporation Solution (Mirus Bio, Madison, WI, USA. #MIR50114). Next, 800 μL of cell suspension was mixed with 5 µg of SARS-CoV-2 full-length RNA and 5 µg of N-gene RNA transcripts, transferred to a 4 mm electroporation cuvette (VWR International, Aurora, CO, USA. #89047-210) and immediately pulsed using a BioRad GenePulser using the exponential protocol (270 V, Capacitance-950 μF, Resistance—∞ (infinity)). Electroporated cells were recovered at room temperature for 5 min before resuspending in a T-75 flask containing DMEM supplemented with 10% FBS and 1X PenStrep. The flask maintained at 37 °C with 5% CO_2_ was regularly monitored, and the virus was harvested when cells exhibited signs of cytopathic effect, typically after 2–3 days. Cells and supernatant were harvested, and the virus stock was referred to as Passage 0 (Figure 1B).

**Figure 2:**
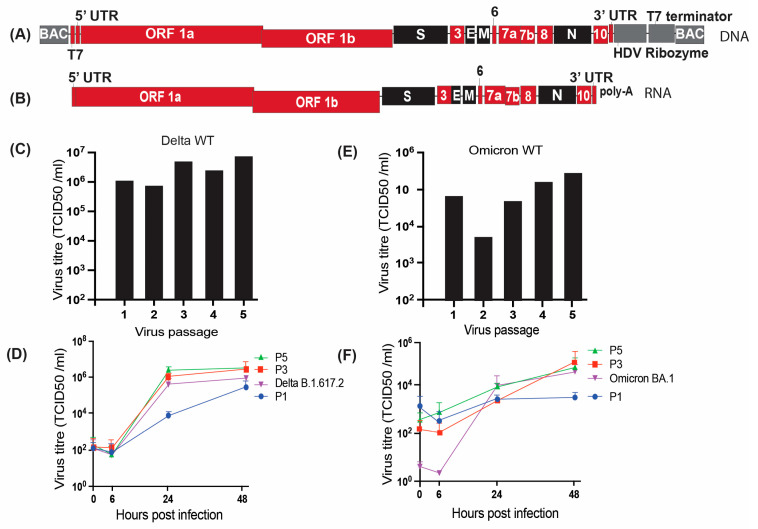
Design and characterization of SARS-CoV-2 Delta and Omicron WT molecular clone. (**A**) Schematic representation of the full-length SARS-CoV-2 WT molecular clone DNA, featuring a T7 promoter, 5′ and 3′UTRs, open reading frames (ORFs), structural genes, poly(A) tail, hepatitis delta virus ribozyme, and T7 terminator within a pCC1BAC vector. (**B**) Schematic representation of the SARS-CoV-2 RNA genome. (**C**) Titres of Delta WT virus stocks (passages 1–5) (TCID/mL). (**D**) Growth curves of SARS-CoV-2 Delta WT virus in Vero cells. (**E**) Titres of Omicron WT virus stocks (passages 1–5). (**F**) Growth curves of SARS-CoV-2 Omicron WT virus in Vero cells. Statistical comparison was performed using one-way ANOVA. Data are presented as the mean ± SD of three independent biological replicate experiments.

Virus growth and passage—Vero cells were used to amplify the cloned virus stocks (Figure 2C) and published primary isolates [18]. To generate Passage 1 of cloned viruses, 500 µL of virus stock from Passage 0 (P0) was used to infect an 80–90% confluent T75 flask of Vero cells, and the supernatant (8 mL) containing the virus was harvested, 3–5 days post-electroporation, when the cells started to show CPE. For the preparation of Passage 2 (p2), 500 µL of the p1 virus stock was used to infect a fresh T75 flask of Vero cells. For subsequent passages (3 through 5), 1 mL of virus harvested from the preceding passage was used as the inoculum. For published primary isolates Delta VOC (B.1.617.2) GenBank Accession #OM131551.1 and Omicron VOC (BA.1) GenBank Accession #OM131552.1 variants, the stocks were obtained from Dr. Darryl Falzarano’s lab at the Vaccine and Infectious Disease Organization (VIDO) [18], and propagated in Vero cells up to Passage 5 as described above. All infections were carried out at 37 °C. All the experiments involving infectious SARS-CoV-2 viruses were conducted at VIDO-Intervac in an approved Biosafety containment level 3 (BSL3) laboratory.

Virus Growth Curves—Vero cells were used to generate virus growth curves to measure the growth kinetics for all clones and primary isolates. One day prior to infection, 1 × 10^4^ Vero cells were seeded/well in a 96-well plate and then infected with virus at an MOI of 0.1 in 50 μL/well of DMEM supplemented with 2% FBS. The virus was adsorbed for 1 h at 37 °C. The virus inoculum was removed and replaced with 200 μL/well of complete media (DMEM supplemented with 10% FBS) and incubated for the next 48 h. 50 μL/well of supernatant was harvested at 0 h, 24 h, and 48 h post-infection and titrated to evaluate virus growth over time. For growth curves based on Nluc expression, 50 μL of supernatant was harvested at 0, 6, 24, and 48 h post-infection and frozen in a white 96-well plate (Corning, Tewksbury, MA, USA, #C3610). The Nluc expression was measured using the Nano-Glo^®^ luciferase assay system (Promega, Madison, WI, USA, #N1120) after equilibrating the 96-well plate at room temperature for 10 min. Then, 50 μL volume of the Nano-Glo^®^ luciferase assay substrate was added to each well, and the plates were read using the Promega^TM^ GloMax ^®^ Explorer (Promega Corporation, Madison, WI, USA # GM3510) plate reader with 5 sec of integration time.

Virus titration by TCID_50_—Virus titres were determined using the 50% tissue culture infectious dose (TCID_50_) method in Vero cells, as previously [11]. Briefly, Vero cells were seeded at 1 × 10^4^ cells per well in 96-well plates. One hundred μL of ten-fold serially diluted virus samples prepared in DMEM supplemented with 2% FBS were added to eight wells each, and cytopathic effect (CPE) was evaluated at 3–5 days post-infection. The virus titre per mL was calculated using the approach of Hierholzer & Killington, with the TCID_50_ calculator [19].

Antiviral compounds—Remdesivir (#HY-104077), Molnupiravir (#HY-135853), and Nirmatrelvir (#HY-138687) were purchased through MedChemExpress (MedChemExpress LLC, Monmouth Junction, NJ, USA). All the drugs were reconstituted in DMSO, aliquoted, and stored at −80 °C until use.

Antiviral Assays—We evaluated three U.S. Food and Drug Administration (FDA)-approved drugs, Remdesivir, Nirmatrelvir, and Molnupiravir, for inhibition of our wild-type and recombinant reporter viruses and published primary isolates in Vero cells. For these assays, Vero cells were cultured in 96-well plates at 1 × 10^4^ cells/well, and the following day, they were infected with 0.1 MOI of virus in 50 μL. The infections were incubated for one hour at 37 °C, then washed twice with PBS, and then incubated with drug/carrier containing DMEM supplemented with 10% FBS. The drugs were diluted to 200µM with fresh media to make a 100 µL volume and further serially diluted 5-fold to 13 nM to generate a dose–response curve. Seventy-two hours post-infection and drug treatment, the supernatants were harvested and assayed for Nluc expression. The time points were determined based on previous studies [11]. The concentration of a drug that gives half-maximal response (IC_50_) was calculated for all the drugs tested using GraphPad Prism 10 software by nonlinear regression. The virus inhibition was normalized to DMSO-treated infected cells, representing 100% virus infection in luciferase fold expression.

Cytotoxicity Assay—To assess the cell viability of Vero cells, a colorimetric cell cytotoxicity (MTS) assay was performed using CellTiter 96^®^ AQueous One Solution Cell Proliferation Assay kit (Promega Corporation, Madison, WI, USA #G3582). The assay was carried out on 96-well microtiter plates cultured at 1 × 10^4^ cells/well. 24 h following cell seeding, the cells were incubated with reconstituted Remdesivir, Nirmatrelvir and Molnupiravir diluted in DMSO, or in DMSO alone. The plates were incubated for 72 h post-drug treatment, and 30 µL supernatant was harvested at 72 h. In a separate 96-well microtiter plate, equal volumes of the CellTiter 96^®^ AQueous One Solution Reagent were added to the supernatant, and the plate was incubated for 2 h in a humidified incubator at 37 °C with 5% CO_2_. The absorbance at 490 nm was measured using a GloMax 96 microplate luminometer. The 50% cytotoxicity concentration (CC_50_) was determined using a dose–response curve plotted to calculate the drug concentration at which the number of viable cells was 50% concentration. The cell viability was normalized to untreated cells, representing 100% cell viability. The CC_50_ value was calculated with GraphPad PRISM10 using the nonlinear regression analysis.

Primary cell culture and infection—For NHBE cell infection, 24-well plates were first coated with collagen. For this, 150 µL of Collagen I, Bovine (Gibco, Grand Island, NY, USA #A1064401) was mixed with 25 mL PBS to make a stock solution. Then, 500 µL of the collagen stock solution was added to each well and incubated for 1 h at 37 °C. The collagen solution was discarded, and the plate was seeded with 5 × 10^4^ NHBE/well in a BEBM media and covered with an aluminum foil as the media is light sensitive and incubated at 37 °C and 5% CO_2_. The following day, 1 MOI of Omicron NLuc virus passage 5, and 0.1 and 1 MOI of Delta NLuc were diluted in BEBM media. The cells were infected for 1 h at 37 °C, and then the inoculum was discarded and replaced with 1 mL/well of fresh media and incubated for the next 48 h. 50 μL/well of supernatants was harvested at 0 h, 24 h, and 48 h post-infection to be used to assay for Nluc expression.

Sequencing—To sequence virus clones, infected cells from 3 wells of a 96-well plate were harvested, and total RNA was extracted using the RNeasy Kits (Qiagen, Hilden, Germany #74106) following the manufacturer’s instructions. Purified RNA samples were processed and full-length SARS-CoV-2 virus RNA sequenced at the Sunnybrook Health Science Centre using the ARTIC SARS-CoV-2 amplification protocol as described [20,21,22]. The RNA whole genome assembly of reads, short-read sequence processing, and Pangolin lineage assignment were performed using the SIGNAL v1.5.6 pipeline (SARS-CoV-2 Illumina Genome Assembly Line), accessible at https://github.com/jaleezyy/covid-19-signal (accessed on 19 November 2025) [21]. The assembled sequences were aligned with reference sequences synthesized by Telesis Bio by using SnapGene Viewer (SnapGene, Chicago, IL, USA). Mutations were identified using SnapGene Viewer’s tools section. The software highlighted single-nucleotide polymorphisms (SNPs) and insertions/deletions (indels) relative to the reference genome.

Statistical Analysis—Unless otherwise stated, all experiments were performed in three independent biological replicates. The data analysis was performed using GraphPad Prism10 software. The data was expressed in Standard Deviation (SD) format. To compare the variability of viral titers at 48 h among Delta and Omicron virus stocks, standard deviations (SDs) were calculated for each group. Differences between groups were assessed using one-way ANOVA. Statistical analyses were performed using GraphPad Prism10 software (GraphPad Software, LLC, San Diego, CA, USA), and *p*-values < 0.05 were considered statistically significant.

## 3. Results

### 3.1. Generation of Wild-Type SARS-CoV-2 Delta and Omicron Molecular Clones

Molecular clones of SARS-CoV-2 Delta and Omicron were designed as full-length cDNA copies in bacmids with a T7 promoter upstream of the virus 5′UTR to generate an authentic 5′ terminal sequence. The viral DNA clone was also flanked by HDV ribozyme sequences followed by T7 terminator sequence, precluding plasmid linearization prior to in vitro transcription (IVT) (Figure 1). The cDNA sequences for Delta and Omicron variants were derived from online sources available at the time of the initiation of each variant (EPISET Identifiers for Delta-3162224, 3162387, 3162814 and for Omicron 249358, 2B95A89, 32703755, 211129-001, VM21044713-1, RIVM-71082, K032193, K032194, K032221, N21665, N21676). Approximately 5 μg of SARS-CoV-2 cDNA bacmid plasmid (synthesized by Telsus Bio) was used to generate in vitro transcribed viral genomic RNA by IVT (Figure 1A,B). The transcribed viral RNA was then electroporated along with an mRNA expressing SARS-CoV-2 Nucleocapsid (N) protein into Vero cells to initiate an infection (Figure 1). Co-transfection of the N gene was previously reported to enhance the successful rescue of the virus in coronavirus reverse genetic systems [23]. For all viruses, CPE was observed 3–5 days post-electroporation, characterized by cell rounding and detachment. The supernatant (called passage 0) was harvested after visual CPE and used to make 5 subsequent virus passages and virus stocks called passage 1 to 5 as described previously. For each virus, we assessed virus titres from all passages (Figure 2C,E), growth curves for passages 1, 3, and 5, and compared them to the published primary isolate of the respective virus (Figure 2D,F). The genome stability after passage in cell culture was determined by whole genome sequencing [12].

### 3.2. Characterization of the Molecular Clone Derived Wild-Type Delta Virus

For the Delta WT virus molecular clone-derived stock, we used a plasmid generated in our lab by growth in bacteria and maxi-prep (Figure 2A) [16]. The authenticity of the plasmid sequence was confirmed by nanopore sequencing (Plasmidsaurus) before generating viral genomic RNA by IVT. Titres of Delta wild-type virus derived from the molecular clone varied from passages 1 through 5, with an increase from 6.8 × 10^5^ TCID_50_/mL at passages 1 and 2 to 4.7 × 10^6^ TCID_50_/mL at passages 3–5 (Figure 2C). Growth curves showed similar growth kinetics for passages 3 and 5 and the published primary isolates Delta B.1.617.2, and a delayed kinetics for passage 1 virus (Figure 2D). The variability of viral titers at 48 h, as measured by SD, did not differ significantly between P1, P3, P5, and Delta B.1.617.2 virus stocks (one-way ANOVA, *p* = 0.3330). The viral genome sequencing identified a single point mutation in the spike at p5 and no change in viral sequence from passage 1 to 4.

### 3.3. Characterization of the Wild-Type Omicron Virus Derived from a Molecular Clone

Using the Omicron wild-type genomic clone, we generated virus stocks for passage 0 to 5. The titres of the stocks in passages 1 to 5 ranged from 3.6 × 10^3^ TCID_50_/mL at passage 2 to 2.04 × 10^5^ TCID_50_/mL in passage 5 (Figure 2E). The virus titres were (4.8 × 10^4^ TCID_50_/mL) at passage 1, then dropped at passage 2 and increased from passages 3 through 5. In addition, there was some evidence of virus sequence instability and possible adaptation to cell culture since passage 4 and 5 virus stocks had mutations that led to amino acid changes in ORF1, NSP5, Spike, and Envelope (Table 1). However, p1, p3, and p5 stocks exhibited similar growth kinetics as published primary isolates in growth assays (Figure 2F). The variability of viral titers at 48 h, as measured by SD, did not differ significantly between P1, P3, P5, and Omicron BA.1 virus stocks (one-way ANOVA, *p* = 0.7236).

### 3.4. Generation of SARS-CoV-2 Delta and Omicron Nano Luciferase Reporter Molecular Clones

**Figure 3:**
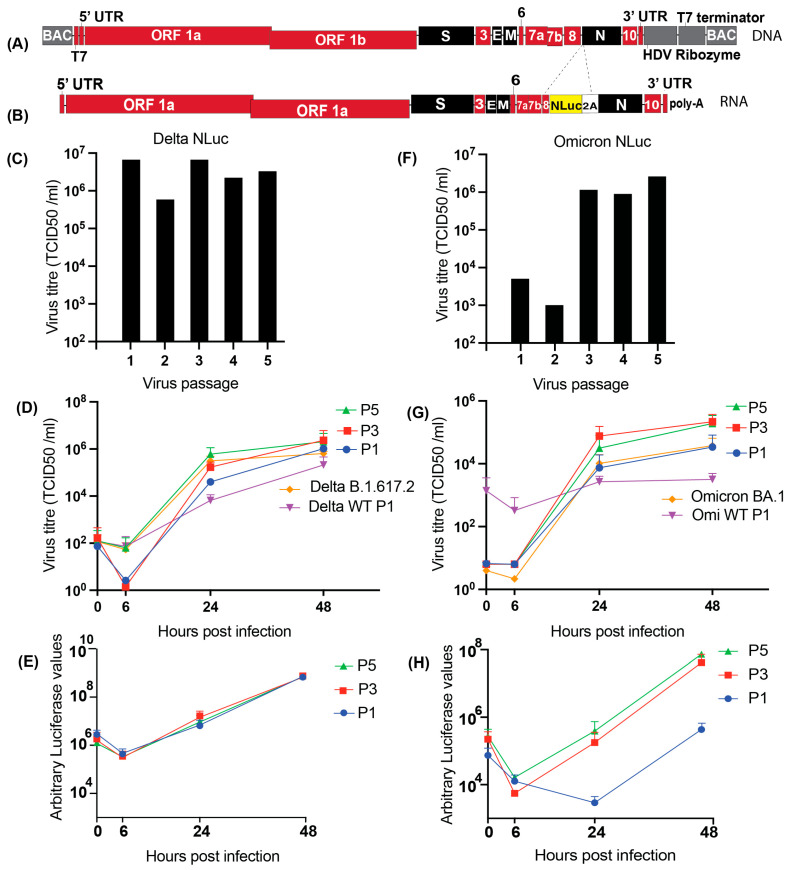
Design and characterization of SARS-CoV-2 Delta Nano-luciferase molecular clone (**A**) Schematic representation of the full-length SARS-CoV-2 WT molecular clone DNA, and (**B**) SARS-CoV-2 NLuc RNA genome. (**C**) Titres of Delta NLuc virus stocks (passages 1 to 5), (**D**) growth curves of the SARS-CoV-2 Delta NLuc virus in Vero cells assessed by virus titre, and (**E**) Nluc expression. (**F**) Titres of Omi NLuc stocks (passages 1 to 5) (**G**) Growth curves of the SARS-CoV-2 Omi NLuc virus in Vero cells assessed by virus titre and (**H**) Nluc expression. Statistical comparison was performed using one-way ANOVA. Data are presented as the mean ± SD of three independent biological replicate experiments.

To make Nluc reporter clones and viruses for Delta and Omicron variants, the Nluc gene was cloned as a fusion with the SARS-CoV-2 N gene separated by a porcine teschovirus-1 2A sequence such that both Nluc and N will be made from the N sgRNA (Figure 3A). Viral RNA (Figure 3B) and viruses were generated as described previously. The P0 stock was amplified for five passages as the titres shown in Figure 3C (Delta Nluc) and F (Omicron Nluc). The titres of Delta NLuc passages ranged from. 4 × 10^5^ TCID_50_/mL to 4.7 × 10^6^ TCID_50_/mL (Figure 3C) and growth curves for passage 1, 3, and 5 analyzed based on virus titres of Delta Nluc showed similar growth kinetics as Delta B.1.617.2 (Figure 3D). Quantitative comparison of viral titer variability at 48 h, using standard deviation as a measure, revealed no significant differences between P1, P3, P5, Delta WT, and Delta B.1.617.2 virus stocks (one-way ANOVA, *p* = 0.7157). In addition to the growth assays based on virus titre, we also assessed Delta Nluc growth based on secreted Nluc expression, and the kinetics were identical for passages 1, 3, and 5 (Figure 3E), similar to the growth curves generated based on virus titres. This supports the use of Nluc as a proxy to measure virus replication efficiency. Sequencing of Delta Nluc viruses at passages 1 and 5 confirmed the stability of the virus sequence. By contrast, Omicron Nluc virus stocks exhibited delayed CPE, 5 days post-infection. We again saw lower titres for passage 1 and 2 stocks and an increase at passage 3, specifically an almost 3-log increase from 5.4 × 10^2^ TCID_50_/mL at passage 2 to 1.4 × 10^6^ TCID_50_/mL at passage 3 (Figure 3F). While passages 2–5 of the Omicron NLuc virus accumulated amino acid substitutions in NSP12 and NSP13 (the viral polymerase and helicase, respectively), as well as in the E and M proteins (Table 1). In addition, passages 4 and 5 had an additional amino acid change in S (Table 1), but this did not appear to modify virus growth kinetics of passages 4 and 5 (Figure 3G,H).

### 3.5. Nano-Luciferase SARS-CoV-2 Reporter Viruses as a Drug-Screening Tool

**Figure 4:**
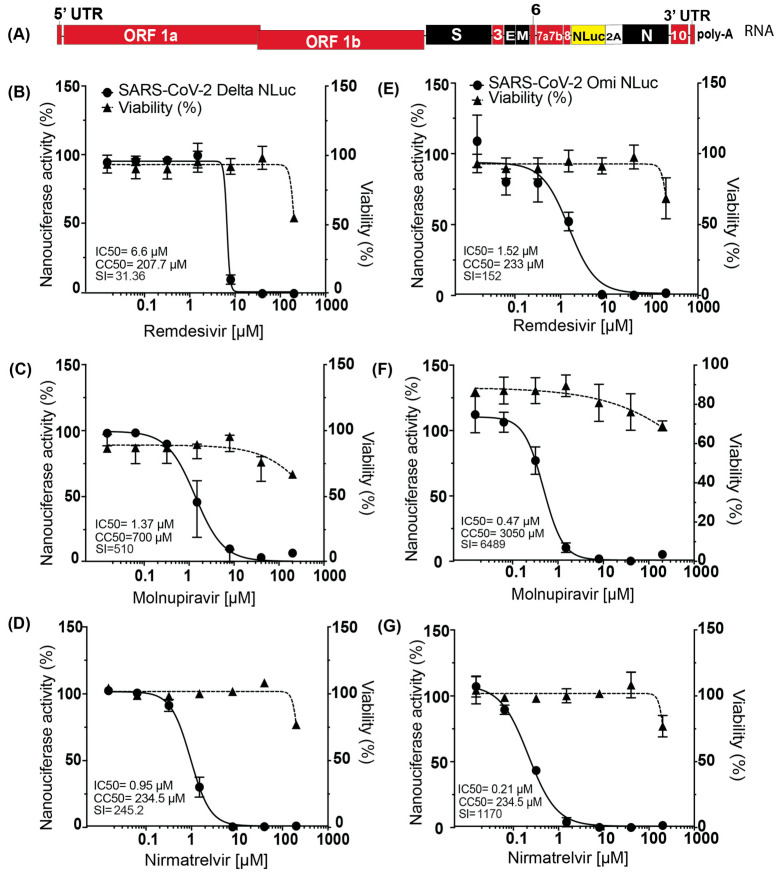
Utility of SARS-CoV-2 Delta and Omicron Nano-luciferase viruses as a drug screening tool. (**A**) Schematic representation of the SARS-CoV-2 NLuc RNA genome. Dose–response curve illustrating the antiviral effect of (**B**) Remdesivir, (**C**) Molnupiravir, or (**D**) Nirmatrelvir on Delta NLuc. Dose–response curve illustrating the antiviral effect of (**E**) Remdesivir, (**F**) Molnupiravir, or (**G**) Nirmatrelvir on Omicron NLuc. The data represent the mean and standard deviation of three biological replicate experiments. IC_50_, CC_50_, and SI values were calculated using GraphPad software.

To test the utility of Delta Nluc and Omicron Nluc in antiviral assays that use Nluc as a proxy for virus replication, we assess their inhibition by known SARS-CoV-2 antiviral drugs Remdesivir, Molnupiravir, and Nirmatrelvir [24] (Figure 4A). To determine the half-maximal inhibitory concentration (IC_50_), Vero cells infected with Delta NLuc or Omicron Nluc were treated with 13 nM, 64 nM, 320 nM, 1.6 μM, 8 μM, 40 μM, and 200 μM concentrations of each drug or with DMSO carrier control, and the secreted Nluc was collected and assayed from the supernatant at 72 h post-treatment. Figure 3B–D show the dose–response curves of the Delta Nluc reporter virus treated with the antiviral drugs. The IC_50_ values for the treatment of Delta Nluc were determined to be 6.6 μM for Remdesivir, 1.37 μM for Molnupiravir, and 0.954 μM for Nirmatrelvir. For Omicron Nluc, the IC_50_ values were 1.537 μM for Remdesivir, 0.47 μM for Molnupiravir, and 0.215 μM for Nirmatrelvir. Our results are consistent with published IC_50_ ranges, including Remdesivir in Vero cells (0.77–23.15 µM) [25,26], Molnupiravir showing an IC_50_ of 0.3–3 µM in Vero cells [27], and Nirmatrelvir with an IC_50_ of 2.128 µM in Vero cells [28,29]. Importantly, the antiviral activity observed using the newly generated Delta and Omicron clones is consistent with our earlier findings using the Wuhan SARS-CoV-2 isolate [11], where Remdesivir showed dose-dependent inhibition in Vero cells across a nanomolar-to-micromolar range (13 nM–200 µM). Cytotoxicity assays were carried out simultaneously and used to calculate the 50% cell cytotoxicity, CC_50_ and selectivity index (SI = CC_50_/EC_50_) for each drug against each virus. The CC_50_ values were measured as follows: 207.7 μM for Remdesivir, 700 μM for Molnupiravir, and 234.5 μM for Nirmatrelvir. Based on the IC_50_ and CC_50_ values, we calculated the selectivity index for Delta Nluc of 31.36 for Remdesivir, 510 for Molnupiravir and 245.2 for Nirmatrelvir. For Omicron Nluc, the SI values were 152 for Remdesivir, 6489 for Molnupiravir and 1170 for Nirmatrelvir. A compound with an SI of 10 or higher is considered safe [30]. These results indicated that the Omicron and Delta reporter NLuc viruses can be used as a tool to screen and analyze the antiviral efficacy and safety of antiviral drugs.

### 3.6. Comparative Growth Kinetics of SARS-CoV-2 Delta Variant in Primary Cells Using Nano-Luciferase Assays

**Figure 5:**
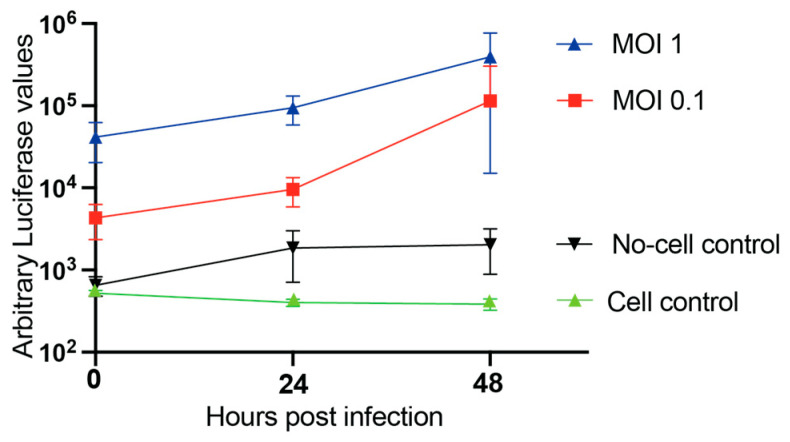
Growth kinetics of SARS-CoV-2 Delta Nluc in primary cells. The growth curves of the SARS-CoV-2 Delta NLuc virus in NHBE infected with an MOI of 0.1 and an MOI of 1, compared to an uninfected cell control and a no-cell-empty-well control. The results are expressed as the mean luciferase values ± SD from three independent biological replicate experiments.

To verify that the Nluc viruses can also be used in replication assays and drug screens in primary cells, we infected NHBE with Delta and Omicron Nluc viruses and assayed replication based on Nluc expression. To test for replication, NHBE were infected with Omicron Nluc at an MOI of 1 and Delta Nluc at an MOI of 0.1 and 1, and virus replication was examined based on Nluc expression. Omicron Nluc did not show an increase in Nluc expression along the time course, indicating a lack of efficient infection or replication of these cells (Figure 5). For Delta Nluc, we observed an increase in Nluc levels at both 24 and 48 h for infections at both MOIs used, compared to a ‘no-cell control’ well in which virus stocks were added to an empty well to detect background levels of Nluc that may have been introduced from the virus stock during the initiation of infection. A 1.5 to 2.0 log increase in Nluc expression in infections initiated by using a Delta Nluc suggests replication in NHBE.

## 4. Discussion

This research involved the synthesis of full-length molecular clones of SARS-CoV-2 Delta and Omicron wild-type and Nluc reporter versions and their use to generate infectious and reporter viruses. Although other plasmid-based SARS-CoV-2 reverse genetic systems have been documented [31,32], this report highlights the use of DNA synthesis and assembly services to rapidly generate single-bacmid-based cDNA clones and reporter viruses. SARS-CoV-2 remains a significant global challenge, and limited treatment options for severe COVID-19 disease make efficient screening tools for the identification of effective anti-SARS-CoV-2 therapeutics important. In addition, new coronaviruses could emerge in the future, and our work shows that DNA synthesis is a viable and rapid option to generate reverse genetic systems for wild-type and reporter viruses using sequence information alone. We previously reported the use of a reverse-genetics system to generate an Nluc reporter virus for the ancestral virus Wuhan-1 by replacing the ORF7A gene with Nluc [11]. The current reporter versions for Delta and Omicron are improved in that they retain the ORF7A open reading frame and confirm that the Nluc gene can be expressed as a 2A fusion with the N gene. This improvement enables the use of these reporter viruses in pathogenic studies in animal models where the ORF7A gene may play a role. In addition, this report confirms that SARS-CoV-2 bacmids can be amplified in bacteria, since the wild-type Delta viruses were derived from bacmids that had been expanded in bacteria to produce them at scale [16].

We next discuss the replication kinetics of the Delta and Omicron variants observed in this study. In the virus growth kinetics performed using TCID_50_, the reduced inoculum volume (0.5 mL) used for passages 1 and 2, compared to 1 mL for passages 3–5, may have partially contributed to a lower viral titre in passages 1 and 2 relative to passages 3–5. Thus, the delay in replication kinetics of the passage 1 stock may have been due to less efficient infections initiated by the lower volume of inoculum used and may have been influenced by the inhibitory activity of cellular material in P0 stock released into culture media from dead cells created by electroporation. The NLuc reporter used in this study is a secreted luciferase, and its activity is continuously released into the culture supernatant throughout infection. Therefore, the NLuc signal serves as a sensitive surrogate readout of viral replication dynamics rather than a direct measure of the number of virions produced per infected cell. To validate that NLuc activity correlates with productive infection, infectious virus yields were quantified in parallel using TCID_50_ assays, which provide a direct measurement of replication-competent virus. Replication and sequence analysis indicate that most of the clone-derived viruses replicated with similar efficiency as wild-type viruses and were genetically stable across 5 passages. However, the titres of Omicron Nluc increased ~3 logs after passage 2. This may have been evidence of adaptation since increased titres correlated with sequence changes in the viral RNA-dependent RNA polymerase (NSP12) and helicase (NSP13), N and E genes that may have enhanced virus fitness as observed in Figure 3F. However, the increase in titres could also have been due to inefficient rescue of the virus from RNA electroporation leading to low titres during several early virus passages until enough virus accumulates to initiate an efficient infection, or due to different cellular responses that may enhance infection efficiency during one passage. In addition, the fitness of Nluc reporter viruses may have been affected by the efficiency of separation of N from the N-terminal fusion with Nluc by the porcine teschovirus-1 2A sequence. The porcine teschovirus-1 2A sequence induces ribosome skipping during translation that causes ‘cleavage’ and separation of the Nluc and the N proteins, but the 2A sequence can be inefficient depending on the context and cell lines used. Since N is required for both virion assembly and efficient SARS-CoV-2 genome replication, lower levels of fully processed N may impair virus fitness and pressure the adaptation of Omicron NLuc to improve replication efficiency, or M and E to pressure efficient virion assembly. We did not observe adaptation in Delta NLuc, but the higher replication efficiency of Delta may have compensated for any deficiencies caused by the Nluc-2A-N fusion in this context.

Antiviral assays with Remdesivir, Molnupiravir, and Nirmatrelvir showed varying inhibitory efficacy against Delta NLuc and Omicron NLuc with minimal toxicity [33]. We also observed an enhanced sensitivity of Omicron to drug inhibition, consistent with previous reports [34]. Delta NLuc could infect primary human bronchial epithelial cells, providing evidence that the virus can infect cells that closely mimic natural human cells. Infection with Omicron-Nluc was unsuccessful in primary cells, likely due to slower replication kinetics and Omicron’s tendency to replicate in the upper respiratory tract cells. These findings enhance the relevance of our research by offering insights into viral behaviour in primary cell line infections and assessing treatment effectiveness in a more representative human model.

It is important to acknowledge that the novel approach described in this study—generating replication-competent viruses from synthetic DNA while introducing heterogeneous gene functions—can be used for “gain-of-function” research. We emphasize that this methodology is strictly intended for controlled laboratory studies to investigate viral biology and gene function and should not be used to create novel viruses with increased pathogenicity, transmissibility, or other potentially hazardous properties. All work was conducted under the highest biosafety standards, in compliance with relevant national and international regulations governing gain-of-function research. The ethical implications of this strategy are recognized, and the study underscores the responsibility of researchers to limit its application to fundamental research rather than the generation of viruses that could pose a threat to public health.

In summary, these full-length infectious genomes show that modern DNA synthesis methods are a viable choice for the rapid generation of reverse genetic systems for coronaviruses, and likely other viruses. This provides a rapid method to study viruses that may emerge and cause outbreaks in the future. We have also shown that NLuc reporter viruses can be used for rapid replication assays, using secreted luciferase as a proxy for viral replication, and serve as a tool for drug testing and therapeutic development. Compared to traditional viral titres, the luciferase readout method simplified the process and provided faster quantification. We have demonstrated that most of our clone-derived viruses replicated similarly to clinical strains, allowing them to serve as effective substitutes and/or models for replication studies. Gene synthesis can also be used to generate mutant viruses to explore and characterize the impacts of specific adaptive mutations that may have evolved in viral variants, to determine their impact on viral fitness, and to better understand virus evolution. The use of modern gene synthesis methods to generate reverse genetic systems to test virus replication, drug screening, and variant evolution makes it an important asset in the ongoing efforts to combat COVID-19 and other viral disease outbreaks in the future.

## Figures and Tables

**Table 1:** Sequence changes identified in virus passaged stocks.

Virus, Mutation and Passage	Nucleotide Position	Nucleotide Change	Amino Acid Change	Viral Gene	p2	p3	p4	p5
Delta WT	21,802	T-A	Asn-Lys	Spike				x
Delta Nluc	284	A-G	Ser-Gly	NSP3	x	x		x
	3643	A-C	No change	NSP3	x	x		x
	26,273	C-T	ser-Leu	E	x	x		x
Omicron WT	348	A-G	Gln-Arg	NSP1			x	x
	10,949	T-A	Phe-Ile	NSP5			x	x
	24,752	G-A	Val-Met	S			x	x
	26,258	C-T	Ser-Leu	E			x	x
Omicron Nluc	7345	A-C	No change	NSP4	x	x	x	x
	13,579	A-G	Thr-Ala	NSP12	x	x	x	x
	17,457	A-G	No change	NSP13	x	x	x	x
	17,466	A-G	No change	NSP13	x	x	x	x
	17,483	A-G	Lys-Arg	NSP13	x	x	x	x
	26,330	C-G	Thr-Arg	E	x	x	x	x
	26,844	A-C	Met-Leu	M	x	x	x	x
	23,684	A-G	Asn-Asp	S			x	x

An ‘x’ indicates that the specific mutation is present in the stated passage number.

## Data Availability

The reagents presented in this study are available upon request from the corresponding author.

## Abbreviations

The following abbreviations are used in this manuscript:

COVID-19	Coronavirus disease 2019
CC_50_	50% Cytotoxicity Concentration
TCID_50_	50% Tissue Culture Infectious Dose
ARDS	Acute Respiratory Distress Syndrome
ANOVA	Analysis of Variance
BAC	Bacterial Artificial Chromosome
BSL3	Biosafety Level 3
BPE	Bovine Pituitary Extract
BEBM	Bronchial Epithelial Cell Growth Basal Medium
CIHR	Canadian Institutes of Health Research
CO_2_	Carbon Dioxide
cDNA	Complementary DNA
CPE	Cytopathic Effect
DNA	Deoxyribonucleic Acid
DMSO	Dimethyl Sulfoxide
MTS	Dimethylthiazol-Carboxymethoxyphenyl-Sulfophenyl-Tetrazolium
DMEM	Dulbecco’s Modified Eagle Medium
DPBS	Dulbecco’s Phosphate-buffered Saline
EPI	Early Psychosis Intervention
E	Envelope
EDTA	Ethylenediaminetetraacetic acid
FBS	Fetal Bovine Serum
FDA	Food and Drug Administration
GISAID	Global Initiative on Sharing All Influenza Data
EC_50_	Half Maximal Effective Concentration
IC_50_	Half Maximal Inhibitory Concentration
HDV	Hepatitis Delta Virus
hEGF	Human Epidermal Growth Factor
IVT	In vitro transcription
Indels	Insertions/Deletions
M	Membrane
mRNA	Messenger Ribonucleic Acid
μM	Micro molar
MOI	Multiplicity of Infection
Nluc	Nano Luciferase
NSERC	Natural Sciences and Engineering Research Council of Canada
NSP	Non Structural Protein
NHBE	Normal Human Bronchial Epithelial
N	Nucleocapsid
ORF	Open Reading Frame
PBS	Phosphate-buffered Saline
SARS-CoV-2	Severe Acute Respiratory Syndrome Coronavirus-2
SI	Selectivity Index
SNPs	Single Nucleotide Polymorphisms
SET	Spreading Evidence-based Treatment
SD	Standard Deviation
SE	Standard Error
TRS	Transcription Regulatory Sequence
UTR	Untranslated Region
VIDO	Vaccine and Infectious Disease Organization
VOC	Variants of Concern
WT	Wild-Type
WHO	World Health Organization
